# Association between glycated hemoglobin and biomechanical properties of the skin with type 2 diabetes by using an electro-mechanical skin device

**DOI:** 10.3389/fendo.2025.1580193

**Published:** 2025-09-05

**Authors:** Fei Chen, Jiahui Zhou, Xianxian Chen, Xuchen Deng, Jiangfeng Zhou, Xin Pan, Zimiao Chen, Xiaohua Gong

**Affiliations:** ^1^ Department of Endocrinology and Metabolism, The First Affiliated Hospital of Wenzhou Medical University, Wenzhou, Zhejiang, China; ^2^ Wenzhou Medical University, Wenzhou, Zhejiang, China; ^3^ Department of Internal Medicine, The First People’s Hospital of Longwan District, Wenzhou, Zhejiang, China; ^4^ Department of Internal Medicine, The people’s hospital of Pingyang, Wenzhou, Zhejiang, China; ^5^ National Key Clinical Specialty (Wound Healing), The First Affiliated Hospital Of Wenzhou Medical University, Wenzhou, Zhejiang, China

**Keywords:** glycated hemoglobin, type 2 diabetes mellitus, electro-mechanical skin device, skin, skin biomechanical properties

## Abstract

**Objective:**

This study aimed to evaluate the association between glycated hemoglobin (HbA1c) levels and biomechanical properties of the human skin with type 2 diabetes mellitus (T2DM) patients using an electro-mechanical skin device.

**Methods:**

A cross-sectional study enrolled 136 T2DM patients and 190 healthy controls. Biomechanical properties of the skin were measured with an electro-mechanical skin device(Khelometer^®^)at different skin sites (forearm, pretibial, and instep regions). The parameters, including F (Maximum rebound force), M (retention rate of rebound force), R (rise response rate) and H (rebound force hysteresis) by Khelometer^®^, represent biomechanical properties of the human skin.

**Results:**

T2DM patients showed significantly higher F values and lower M, R, and H values compared to healthy controls. HbA1c levels were positively associated with F values and negatively associated with M, R, and H values across different skin sites. Participants with higher F values had significantly higher HbA1c levels. Restricted cubic spline analysis revealed strong nonlinear associations between HbA1c and skin biomechanical properties.

**Conclusions:**

The data indicate the electro-mechanical skin device is useful to quantitate the biomechanical properties of the skin. Furthermore, this study demonstrates a nonlinear relationship between HbA1c levels and the biomechanical properties of the skin. These findings add to the evidence to support the impact of elevated HbA1c on biomechanical properties in individuals with T2DM.

## 1 Background

Type 2 Diabetes Mellitus (T2DM) is a chronic metabolic disorder characterized by insulin resistance and relative insulin deficiency, resulting in hyperglycemia. The global burden of T2DM is projected to escalate significantly in the coming decades. According to the International Diabetes Federation (IDF), the number of adults aged 20–79 years living with diabetes is estimated to rise from 463 million in 2019 to 700 million by 2045, with T2DM comprising the majority of cases. Skin, serving as a vital organ of the human body, exhibits mechanical characteristics including stiffness, thickness, retraction, extension, pliability, elasticity, and viscosity. These mechanical properties are vital for the skin to act as a barrier, protecting the body from external factors and withstanding mechanical stresses induced by physical activities and other influences (1, 2). Over time, in patients with diabetes, high blood sugar levels will damage many biological tissues in the body, leading to disability and life-threatening medical complications (3). Approximately one-third of individuals with diabetes develop skin lesions, making it one of the most prevalent symptoms associated with the condition. Skin complications induced by diabetes typically manifest prior to complications affecting other organs (4). As the most superficial organ of our body, Measuring the changes of skin structural characteristics is an important method for clinical evaluation of skin aging and diabetes complications. Studies have shown that the endogenous processes of skin aging caused by diabetes include excessive production of free radicals, nuclear/mitochondrial gene mutations, cell aging, telomere shortening, decreased cell proliferation, and impaired immune function (5, 6). In recent years, studies have also shown that advanced glycation end products (AGE) are one of the key factors causing skin aging (5, 7, 8). In addition to the accumulation of AGEs, chronic hyperglycemia in diabetes triggers a cascade of biochemical disturbances that contribute to skin damage. One key mechanism involves the binding of AGEs to their specific receptors (RAGE), which activates downstream signaling pathways such as NF-κB and induces the release of pro-inflammatory cytokines including tumor necrosis factor-alpha (TNF-α) and interleukin-6 (IL-6) (9). This sustained inflammatory environment leads to fibroblast dysfunction, extracellular matrix degradation, and impaired collagen remodeling. Moreover, increased oxidative stress further exacerbates tissue injury by promoting lipid peroxidation and DNA damage (10). Another important factor is diabetic microangiopathy, which impairs dermal capillary circulation, resulting in reduced oxygen and nutrient delivery to skin tissues and delayed wound healing (11). Together, these biochemical processes disrupt the structural and functional integrity of the skin, ultimately altering its mechanical properties and increasing susceptibility to injury in diabetic patients. Furthermore, the microstructure of collagen and its molecular tissues is also affected by skin aging (12). The atomic force microscope is employed to detect skin biopsy samples, which reveal that the mechanical properties of collagen in diabetic skin have undergone significant changes (13). These include the destruction of dermal collagen integrity, the confusion of collagen fibers, and the nano-scale fragmentation of collagen fibers.

In the past, some scholars have conducted research on the skin of patients with diabetes. Tahrani et al. evaluated patients with diabetes peripheral neuropathy (DPN) using the Michigan Neurology Screening Instrument (MNSI). Skin biopsy (3mm) was performed on the upper and lower legs skin of subjects to measure intraepidermal nerve fiber density (IENFD), structural analysis, abundance of type 1 procollagen, tissue degrading MMPs, and poly ADP ribose (PAR) immunoreactivity. The study found that the increase of PAR, the decrease of type 1 procollagen abundance and the damage of skin structure were related to the foot complications of diabetes (14). Their measurement method, however, was invasive and lacked the capability to dynamically monitor pathological changes in the skin of the same individual. Viktor Dremin et al. (3)introduced a novel polarization-enhanced neural network-assisted hyperspectral method for monitoring blood content, blood oxygen, and skin structural changes in diabetes mellitus (DM) patients. Xian Wang et al. (15)measured skin spontaneous fluorescence (SAF) using a fluorescence reader to represent the accumulation of AGEs in skin tissue. They proposed that SAF is an independent marker of diabetes retinopathy, diabetes nephropathy, cardiovascular disease and diabetes peripheral neuropathy, and also a predictor of the complexity of T2DM complications. Nevertheless, these methods for assessing the complications of diabetes are complex and difficult to operate. Some rehabilitation clinical practices rely on palpation, which is not an objective parameter for changes in skin mechanical properties (16).

Therefore, employing objective and noninvasive tools to evaluate skin biomechanical properties may provide critical insights into early skin alterations in diabetes. Traditional assessment methods, such as biopsy or visual inspection, are either invasive or subjective, limiting their clinical applicability for routine screening or longitudinal monitoring. The Khelometer^®^(AS-GP1, ASCH Japan Co.,Ltd, Japan), an electro-mechanical device capable of quantifying lateral skin stiffness and elasticity across multiple anatomical regions, offers a novel solution with high reproducibility and sensitivity (17, 18). However, few studies have systematically compared skin biomechanical characteristics between T2DM patients and healthy controls using this device. This study was therefore designed to evaluate the alterations in skin stiffness and elasticity in patients with T2DM compared to healthy individuals, using the Khelometer^®^. By establishing the relationship between glycemic control and skin biomechanical properties, we aim to contribute to the early detection of diabetes-related skin complications and to support the development of personalized preventive strategies in clinical practice.

## 2 Materials and methods

### 2.1 Study population

This cross-sectional study enrolled 136 patients with diabetes in the First Affiliated Hospital of Wenzhou Medical University and 190 healthy subjects in the First Affiliated Hospital of Wenzhou Medical University Physical Examination Center from April to June in 2023. The study was approved by the First Affiliated Hospital of Wenzhou Medical University ethical committee. The diagnostic criteria for T2DM include (1) fasting blood glucose of ≥7.0 mmol/l (2), random blood glucose of ≥11.1 mmol/l or stimulated blood glucose of ≥11.1 mmol/L after a standard oral glucose tolerance test, and (3) glycated hemoglobin (HbA1c) of ≥6.5%. We required all healthy subjects to meet the following characteristics: (1) No previous history of diabetes, (2) fasting blood glucose of ≤6.1mmol/L and stimulated blood glucose of ≤7.8mmol/L after a standard oral glucose tolerance test. Exclusion criteria included: age under 18 years; cancer; known pregnancy; history of liver or kidney insufficiency, or current hepatic/renal dysfunction (alanine aminotransferase (ALT) or aspartate transaminase (AST) > 2× ULN, abnormal total bilirubin, elevated serum creatinine, or estimated Glomerular Filtration Rate (eGFR) < 60 mL/min/1.73 m²); history of heart failure, ejection fraction <50%, or NYHA Class II–IV symptoms; markedly elevated NT-proBNP levels based on age-specific reference ranges; history of venous thrombosis; malnutrition, defined as body mass index (BMI) < 18.5 kg/m², significant weight loss (>5% within 3 months), or low serum albumin levels; overweight or obesity, defined as BMI > 24 kg/m²; and presence of scars, tattoos, rashes, or other skin abnormalities at the testing sites.

The sample size was determined based on previous cross-sectional studies investigating the association between skin biomechanical properties and diabetes status. Although no formal power analysis was conducted, a minimum of 100 participants per group was targeted to ensure adequate statistical power to detect moderate effect sizes (Cohen’s d ≥ 0.5) with 80% power at a 5% significance level. Ultimately, 136 patients with T2DM and 190 healthy controls were enrolled, exceeding this threshold and enhancing the statistical robustness of the findings.

### 2.2 Parameters

The subjects’ age and sex were recorded. Height, weight and blood pressure were measured, and the BMI was calculated as weight (kg) divided by height squared (m^2^).

### 2.3 Laboratory examination

The blood samples were drawn when the subjects were in the morning fasting state and in a sitting position. Lipid metrics, including total cholesterol (TC), high-density lipoprotein cholesterol (HDL-C), low-density lipoprotein cholesterol (LDL-C), and triglycerides (TG), were measured by the standard enzymatic method (AU580 Biochemical Analyzer, Beckman Coulter, Brea, CA, USA). Liver enzymes, including ALT, AST, creatinine and glucose were measured using the standard enzymatic method (AU580 Biochemical Analyzer, Beckman Coulter, Brea, CA, USA). HbA1c was measured using high-performance liquid chromatography (Variant II turbo, BioRad Laboratories, CA, USA) and was expressed in National Glycohemoglobin Standardization Program units (%). While biomarkers such as NT-proBNP could provide additional insights, their absence was offset by strict exclusion criteria for cardiac dysfunction.

### 2.4 Measurement of skin mechanical properties

**Figure 1:**
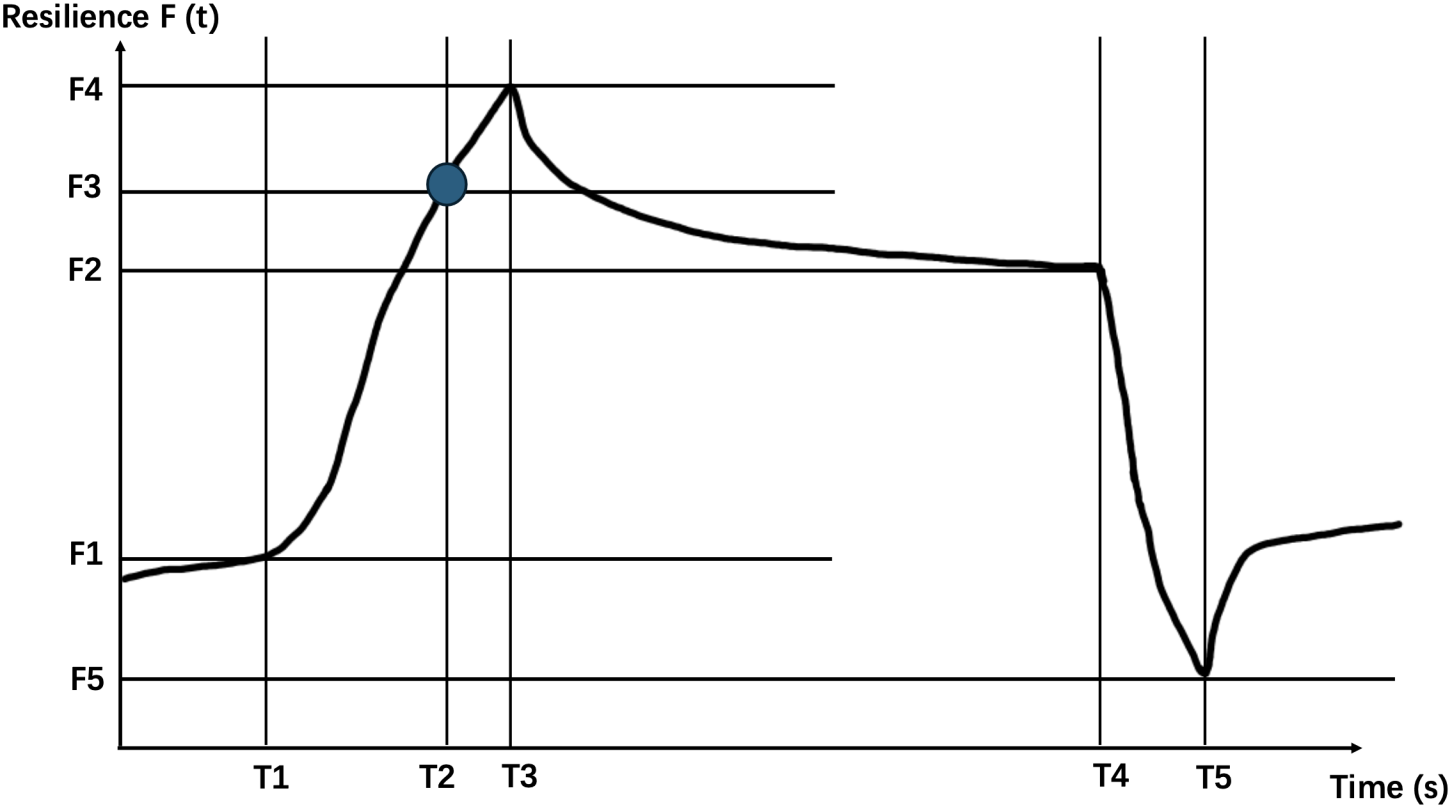
Schematic diagram of the waveform generated by the contact probe of the Khelometer^®^ skin elasticity meter.

The Khelometer^®^ skin elasticity meter (AS-GP1, ASCH Japan Co.,Ltd, Japan) is utilized in our study. By placing the sensor probe on the skin and sliding it across, the device measures elasticity by assessing the stress response of the skin. Specifically, we measure the skin on the forearm, pretibial region, and instep of the participants. The elasticity meter provides the following key data points: (**Figure 1**).

T1: Time of contraction
F1: Rebound force at the beginning of contraction
T2: Time to reach maximum contraction
F2: Rebound force at the beginning of maximum contraction release time
T3: Time to reach maximum rebound force
F3: T2 time rebound force
T4: Time to start releasing contractions
F4: Maximum rebound force
T5: Time to reach minimum rebound force
F5: Minimum rebound force F
(t): Changes in rebound force over time
We further calculate based on the above data using the following formula.

F=F4−F1 (Maximum Rebound Force): This parameter reflects the stiffness of the skin. A higher F value signifies a harder, less pliable skin texture.

$M=\frac{\left(F2-F1\right)}{\left(F4-F1\right)}\cdot 100$ (Retention Rate of Rebound Force): It quantifies the degree to which the maximum rebound force is sustained. A higher M value indicates superior skin elasticity, signifying better ability to retain its shape and resilience upon deformation.

$R=\frac{2\int_{T2}^{T1}\left(F\left(t\right)-F1\right)dt}{\left(T2-T1\right)\cdot \left(F3-F1\right)}\cdot 100$ (Rise Response Rate): This metric signifies the responsiveness of the skin. A lower R value suggests a higher likelihood of skin degradation or reduced ability to quickly recover from deformation.

$H=\left(\frac{\left(F2-F5\right)}{F}-1\right)\cdot 100$ (Rebound Force Hysteresis): It evaluates the lag phenomenon that occurs when a load is applied to the skin. A larger negative H value signifies a more pronounced hysteresis effect, indicative of reduced resilience or a slower return to its original state after deformation.

### 2.5 Measurement procedure

**Figure 2:**
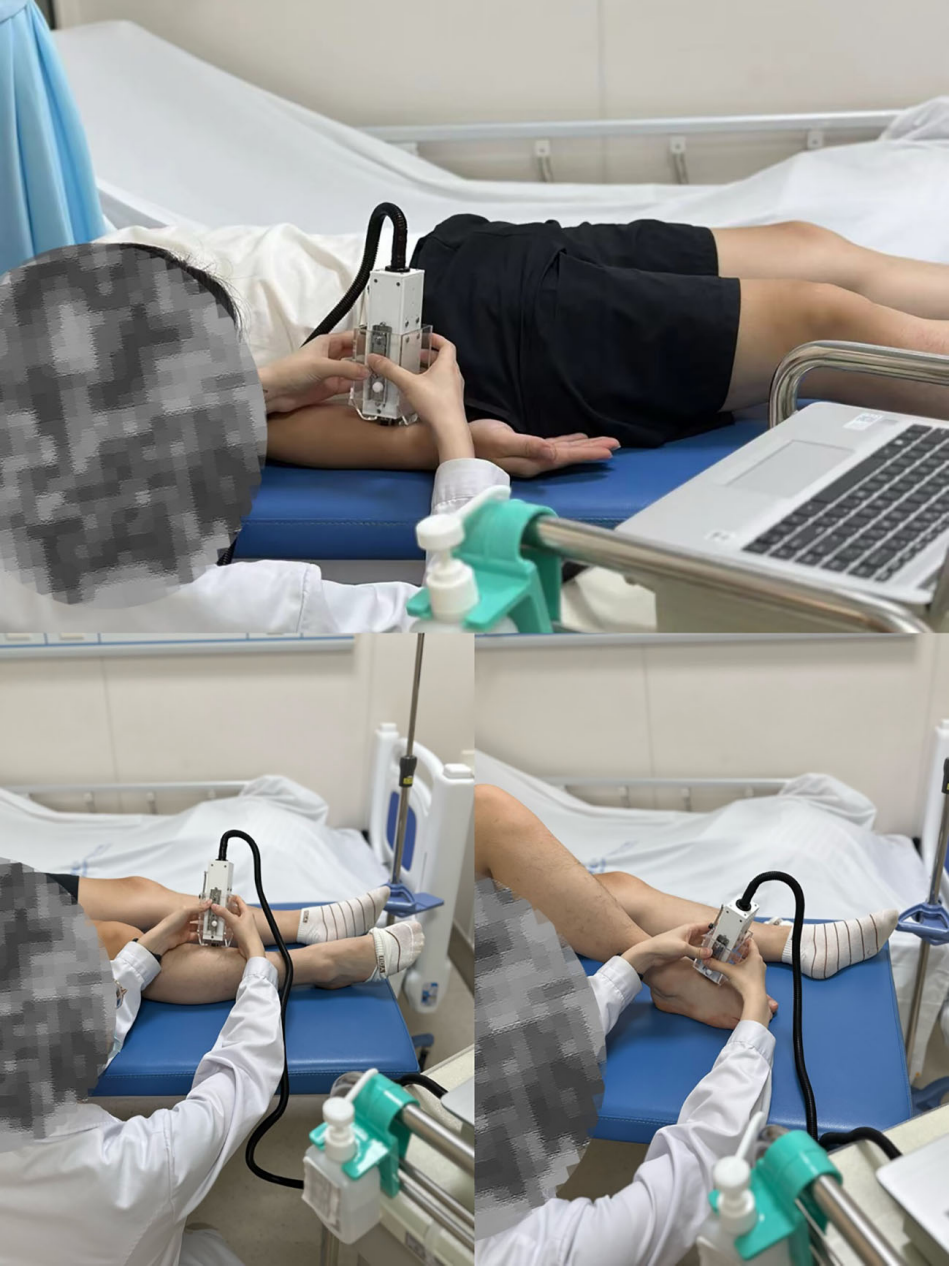
An electro-mechanical skin device (Khelometer^®^) for measuring the biomechanical properties of the skin.

Before embarking on data collection, the tester underwent rigorous training to ensure proficient use of the skin elasticity meter and standardization of the entire measurement process. Throughout the experimental sessions, participants were instructed to adopt a relaxed supine position, with their elbows extended and knees gently bent, maintaining this posture consistently throughout the skin elasticity assessments (as illustrated in **Figure 2**).

The focus of skin data measurements was on three specific anatomical locations: the forearm, the pretibial region, and the instep. These standardized measurement sites were precisely defined as follows:

Forearm: Located 10 centimeters proximal to the transverse carpal tunnel.
Pretibial: Skin directly over the anterior tibia.
Instep: Skin covering the dorsal aspect of the foot.

At each of these sites, three consecutive measurements were taken to ensure reliability, and the mean value of these measurements was calculated for analysis. To eliminate potential bias, the tester was blinded to the results of previous measurements.

The measurements were carried out in a controlled environment, maintaining a temperature of 23 ± 2 °C and a relative humidity within the range of 50% to 60%, ensuring optimal conditions for accurate skin elasticity assessments.

### 2.6 Statistical analysis

The baseline characteristics of the study participants are presented as means with standard deviations (SDs) for normally distributed variables and as median values for non-normally distributed variables. To compare the baseline differences between patients and healthy subjects, we employed the Student’s t-test for normally distributed variables and the Mann-Whitney U test for non-parametric data.

Multivariate linear regression analysis was utilized to estimate the Odds ratio (OR) and their corresponding 95% confidence intervals (CIs) for the associations between HbA1c levels and skin indicators. To account for potential confounding factors, we adjusted these models for age, sex, hypertension status, lower limb atherosclerosis, and carotid atherosclerosis. Following this adjustment, we repeated the linear regression analysis to ensure the robustness of our findings.

Furthermore, we categorized the data into three groups based on the third percentile method to facilitate comparisons across groups. For these categorical variables, we employed analysis of variance (ANOVA) for normally distributed outcomes and the Kruskal-Wallis test for non-parametric data to assess significant differences between the groups.

To characterize the dose-response relationship and explore whether the association between HbA1c and skin indicators follows a linear or nonlinear pattern, we conducted restricted cubic spline analyses (RCS). These analyses provide a flexible way to model continuous variables without assuming a strict linear relationship, allowing us to identify potential thresholds or inflection points in the association.

RCSs was performed using R software version 4.4.0. The SPSS 23.0 software (SPSS Inc., Chicago, IL) was used for other statistical analysis. *P*<0.05 was considered statistically significant.

## 3 Result

**Table 1** presented data comparing patients with T2DM to healthy individuals. Our analysis revealed that T2DM patients exhibited significantly higher levels of BMI, fasting blood glucose, HbA1c, ALT, creatinine, as well as higher F values in the forearm, pretibial region, and instep. Conversely, they showed lower R values in the forearm and instep region, and lower pretibial M and instep H values compared to the healthy subjects.

**Table 1:** Baseline data for all subjects.

	Patients	Normal	Total	*P*
	n=136	n=190	n=326	
Sex (men/women)	82/54	97/93	179/147	0.062
BMI (kg/m2)	27.33±3.00	23.84±2.78	25.30±3.34	**< 0.001**
Age	58.70±13.31	56.56±8.00	57.45±10.57	0.071
Fasting blood glucose(mmol/L)	8.10±3.17	5.00±0.54	6.29±2.59	**< 0.001**
HbA1c(%)	9.96±6.73	5.67±0.32	7.45±4.82	**< 0.001**
ALT(U/L)	31.49±39.42	25.05±16.83	27.73±28.61	**0.045**
AST(U/L)	28.50±26.28	25.61±8.35	26.82±18.13	0.155
Albumin(g/L)	37.51±5.23	39.13±5.26	38.27±5.22	0.141
Creatinine(umol/L)	76.09±51.26	67.96±14.46	71.34±35.01	**0.038**
Forearm F (g)	187.72±44.20	141.89±34.26	161.10±44.82	**< 0.001**
Forearm M (%)	78.26±3.10	77.69±3.81	77.87±3.60	0.228
Forearm R (%)	108.2±3.99	110.92±4.73	109.79±4.63	**< 0.001**
Forearm H (%)	0.09±5.53	1.16±5.86	0.72±5.74	0.096
Pretibial F (g)	206.57±48.81	189.68±45.55	196.45±47.53	**0.004**
Pretibial M (%)	76.60±6.15	80.03±3.36	78.60±5.01	**< 0.001**
Pretibial R (%)	106.85±4.69	107.03±3.74	106.96±4.16	0.703
Pretibial H (%)	0.19±5.38	0.38±5.35	0.30±5.35	0.758
Instep F (g)	195.46±56.61	156.88±47.74	173.05±54.97	**< 0.001**
Instep M (%)	76.17±5.47	76.24±5.63	76.21±5.56	0.905
Instep R (%)	105.68±4.15	107.35±4.68	106.66±4.54	**0.001**
Instep H (%)	-1.44±4.52	0.78±4.91	-0.12±4.88	**< 0.001**

BMI, body mass index; HbA1c, glycated hemoglobin; ALT, alanine aminotransferase; AST, aspartate transaminase. *P* < 0.05 were considered statistically significant. Data with P<0.05 are marked in bold for clarity.

To delve deeper, we conducted multivariate linear regression analyses to explore the relationship between HbA1c levels and various biomechanical properties of skin. Our findings indicated a positive correlation between HbA1c levels and the F values of the forearm, pretibial region, and instep, suggesting that increased HbA1c levels, were associated with increased skin stiffness among T2DM patients. However, our analysis also uncovered negative correlations between HbA1c level and the R value of the forearm, the M and H values of the pretibial region, as well as the R and H values of the instep. Notably, these negative correlations remained significant even after adjusting for potential confounding factors such as age, sex, hypertension, lower limb atherosclerosis, and carotid atherosclerosis (**Table 2**).

**Table 2:** The relationship between HbA1c and skin indicators by multivariate linear regression analysis.

	Uncorrected		Corrected	
	β(95%CI)	*P*	β(95%CI)	*P*
Forearm F (g)	2.453 (1.474,3.431)	**< 0.001**	2.331 (1.341,3.321)	**< 0.001**
Forearm M (%)	0.045 (-0.039,0.129)	0.295	0.045 (-0.040,0.131)	0.297
Forearm R (%)	-0.175 (-0.278,-0.072)	**0.001**	-0.182 (-0.287,-0.076)	**0.001**
Forearm H (%)	-0.050 (-0.179,0.080)	0.453	-0.034 (-0.167,0.098)	0.611
Pretibial F (g)	2.203 (0.125,4.282)	**0.038**	2.094 (0.025,4.164)	**0.047**
Pretibial M (%)	-0.147 (-0.259,-0.034)	**0.011**	-0.133 (-0.246,-0.019)	**0.022**
Pretibial R (%)	-0.016 (-0.110,0.078)	0.731	-0.024 (-0.117,0.070)	0.619
Pretibial H (%)	-0.162 (-0.282,-0.042)	**0.009**	-0.165 (-0.286,-0.044)	**0.008**
Instep F (g)	1.990 (0.762,3.218)	**0.002**	1.808 (0.577,3.038)	**0.004**
Instep M (%)	0.021 (-0.106,0.148)	0.741	0.050 (-0.078,0.178)	0.444
Instep R (%)	-0.110 (-0.212,-0.008)	**0.034**	-0.109 (-0.212,-0.007)	**0.037**
Instep H (%)	-0.280 (-0.491,-0.069)	**0.009**	-0.263 (-0.473,-0.053)	**0.014**

The data on the left is unadjusted, and the data on the right is adjusted. *P* < 0.05 were considered statistically significant. All models were adjusted for age, sex, BMI, fasting blood glucose, total cholesterol, high-density lipoprotein cholesterol, low-density lipoprotein cholesterol, triglycerides concentration, albumin, and creatinine. Data with P<0.05 are marked in bold for clarity.

The participants were stratified into tertiles based on the biomechanical properties of their skin. The group with a high F value (T3) exhibited significantly higher HbA1c levels and fasting blood glucose compared to those with a low F value (T1) and the intermediate group with a moderate F value (T2) (**Tables 3**-**5**). The group with a high R value (T3) of the forearm and instep region exhibited significantly lower HbA1c levels and fasting blood glucose compared to those with a low R value (T1) and the intermediate group with a moderate R value (T2) (**Tables 3**, **5**). The group with a high M value and H value (T3) also demonstrated similar results compared to those groups with T1) and the groups with T2(**Tables 4**, **5**).

**Table 3:** Baseline characteristics of the subjects based on forearm F and R tertiles.

	Forearm F				Forearm R			
	T1	T2	T3	*P*	T1	T2	T3	*P*
	≤140.86 g	[140.86 g,177.80 g]	≥177.80 g		≤107.40%	[107.40%,111.08%]	≥111.08%	
	n=120	n=99	n=107		n=109	n=125	n=92	
BMI (kg/m2)	24.91±3.14	24.93±3.51	26.18±3.30	**0.006**	26.06±3.22	25.09±3.37	24.66±3.31	**0.008**
Age	56.26±10.76	58.38±10.14	58.03±10.81	0.280	58.08±10.95	56.90±11.06	57.42±9.45	0.697
Fasting blood glucose(mmol/L)	5.99±2.43	5.70±1.80	7.25±3.11	**< 0.001**	6.96±2.56	6.31±2.54	6.07±2.69	**0.043**
HbA1c(%)	6.74±2.26	6.62±1.77	9.06±7.72	**< 0.001**	7.99±2.37	7.20±2.27	5.75±2.04	**0.017**
ALT(U/L)	26.19±21.47	29.65±42.30	27.46±19.49	0.687	28.59±19.28	29.94±40.38	23.69±15.51	0.263
AST(U/L)	26.76±16.26	26.24±17.44	27.39±21.06	0.906	27.22±14.84	28.52±24.50	24.01±9.13	0.186
Creatinine(umol/L)	67.25±15.03	69.81±41.51	76.60±43.94	0.124	70.38±27.39	66.62±16.57	78.90±55.04	**0.036**
TC (mmol/L)	5.19±1.92	5.32±1.64	4.80±1.21	0.060	5.14±2.01	5.06±1.37	5.16±1.51	0.901
TG (mmol/L)	2.05±4.02	1.57±0.86	1.76±1.04	0.380	2.13±4.24	1.71±0.95	1.54±0.78	0.239
HDL (mmol/L)	1.29±0.31	1.34±0.29	1.19±0.29	**0.001**	1.25±0.29	1.28±0.31	1.30±0.34	0.586
LDL (mmol/L)	3.27±1.10	3.36±1.25	3.04±0.89	0.098	3.20±1.13	3.23±1.09	3.26±1.08	0.915

BMI, body mass index; HbA1c, glycated hemoglobin; ALT, alanine aminotransferase; AST, aspartate transaminase; TC, total cholesterol; TG, triglycerides; HDL, high-density lipoprotein; LDL, low-density lipoprotein. *P* < 0.05 were considered statistically significant. Data with P<0.05 are marked in bold for clarity.

**Table 4:** Baseline characteristics of the subjects based on pretibial F, M and H tertiles.

	Pretibial F				Pretibial M				Pretibial H			
	T1	T2	T3	*P*	T1	T2	T3	*P*	T1	T2	T3	*P*
	≤177.80 g	[177.80 g,221.77 g]	≥221.77 g		≤77.83%	[77.83%,80.72%]	≥80.72%		≤0.20%	[0.20%,2.40%]	≥2.40%	
	n=104	n=99	n=113		n=112	n=114	n=100		n=110	n=113	n=103	
BMI (kg/m2)	24.88±3.47	25.59±3.29	25.24±3.48	0.368	25.40±3.43	25.57±3.46	24.74±3.15	0.196	25.58±3.29	25.29±3.27	24.93±3.62	0.388
Age	56.39±11.31	57.38±9.86	58.57±10.99	0.390	56.89±10.41	57.64±10.62	57.98±10.24	0.748	58.06±11.08	56.69±10.87	58.01±9.92	0.568
Fasting blood glucose(mmol/L)	5.62±2.80	6.05±2.44	7.39±2.45	**0.027**	7.55±2.91	6.20±2.17	5.89±2.48	**0.019**	7.35±2.62	6.43±2.59	6.02±2.51	**0.025**
HbA1c(%)	6.16±2.32	7.10±8.24	8.27±2.29	**0.014**	7.99±2.46	7.20±2.30	6.08±8.29	**0.008**	8.28±2.37	6.42±2.45	5.7±7.98	0.822
ALT(U/L)	25.18±15.82	29.46±24.79	31.68±44.18	0.343	27.57±39.29	30.75±26.10	23.98±14.92	0.264	25.89±17.83	29.49±41.64	27.78±20.03	0.655
AST(U/L)	26.46±14.45	26.41±14.64	30.01±26.66	0.354	25.45±17.28	28.65±18.43	25.42±12.95	0.271	27.82±20.09	25.81±17.45	26.29±13.93	0.676
Creatinine(umol/L)	70.92±41.65	70.36±25.76	68.28±17.43	0.821	72.51±40.06	70.17±38.58	71.14±26.21	0.891	70.84±38.45	68.59±19.00	75.27±44.52	0.389
TC (mmol/L)	5.38±2.13	4.97±1.45	5.09±1.40	0.242	4.98±1.24	5.30±2.02	5.14±1.68	0.382	5.12±1.45	4.96±1.45	5.3±2.05	0.344
TG (mmol/L)	2.17±4.62	1.64±0.84	1.72±0.91	0.385	1.71±1.10	2.13±4.28	1.59±0.81	0.321	1.69±0.98	1.63±0.91	2.16±4.41	0.280
HDL (mmol/L)	1.30±0.30	1.28±0.34	1.26±0.31	0.604	1.27±0.32	1.31±0.31	1.27±0.31	0.470	1.29±0.31	1.25±0.29	1.3±0.34	0.546
LDL (mmol/L)	3.34±1.19	3.15±1.10	3.24±1.01	0.516	3.13±0.88	3.32±1.17	3.26±1.29	0.472	3.25±1.04	3.15±1.17	3.3±1.12	0.640

BMI, body mass index; HbA1c, glycated hemoglobin; ALT, alanine aminotransferase; AST, aspartate transaminase. TC, total cholesterol; TG, triglycerides; HDL, high-density lipoprotein; LDL, low-density lipoprotein. P < 0.05 were considered statistically significant. Data with P<0.05 are marked in bold for clarity.

**Table 5:** Baseline characteristics of the subjects based on Instep F, R and H tertiles.

	Instep F				Instep R				Instep H			
	T1	T2	T3	*P*	T1	T2	T3	*P*	T1	T2	T3	*P*
	≤147.35 g	[147.35 g,196.31 g]	≥196.31 g		≤104.55%	[104.55%,108.51%]	≥108.51%		≤-1.10%	[-1.10%,1.30%]	≥1.30%	
	n=105	n=105	n=116		n=111	n=108	n=107		n=110	n=109	n=107	
BMI (kg/m2)	25.33±3.56	25.06±3.02	25.54±3.48	0.584	25.48±3.71	25.3±3.22	25.06±3.03	0.641	25.63±3.39	25.49±3.24	24.85±3.37	0.192
Age	57.13±10.62	58.23±10.44	56.9±11.07	0.630	58.29±11.55	56.44±11	57.55±9.07	0.435	57.04±10.24	58.06±11.56	57.02±10.16	0.722
Fasting blood glucose(mmol/L)	6.01±2.66	5.84±2.37	7.23±2.72	**0.021**	7.67±2.90	6.13±2.26	5.89±2.56	**0.032**	7.20±2.20	6.77±2.93	5.96±2.63	**0.027**
HbA1c(%)	6.12±2.57	6.35±7.74	7.67±2.41	**0.011**	7.96±2.36	7.01±2.11	6.82±7.77	**0.025**	8.00±2.26	6.57±2.49	6.47±7.83	**0.020**
ALT(U/L)	25.58±20.08	23.69±15.23	34.33±43.03	**0.018**	34.17±43.47	25.52±18.04	23.74±13.88	**0.016**	26.92±22.53	31.95±42.05	25.18±15.64	0.214
AST(U/L)	27.17±20.78	24.38±8.35	28.97±22.62	0.192	30.47±26.56	26.21±14.44	23.94±7.51	**0.026**	27.16±20.64	28.15±20.69	25.61±12.66	0.602
Creatinine(umol/L)	68.72±14.74	74.15±46.86	72.62±37.22	0.522	69.82±22.34	71.92±47.74	72.43±30.76	0.846	72.02±33.22	67.63±21.04	74.82±47.28	0.336
TC (mmol/L)	5.21±2.09	5.28±1.25	4.79±1.34	0.062	4.80±1.40	5.33±2.06	5.21±1.36	**0.045**	4.84±1.29	5.30±2.07	5.17±1.50	0.115
TG (mmol/L)	2.13±4.35	1.62±0.93	1.63±0.77	0.270	1.73±0.94	2.07±4.26	1.63±0.92	0.420	1.59±0.92	2.25±4.31	1.59±0.89	0.106
HDL (mmol/L)	1.25±0.34	1.37±0.29	1.2±0.29	**< 0.001**	1.21±0.31	1.30±0.31	1.32±0.31	**0.028**	1.25±0.31	1.26±0.32	1.31±0.30	0.407
LDL (mmol/L)	3.28±1.18	3.31±0.94	3.04±0.97	0.111	3.04±1.08	3.36±1.20	3.29±0.99	0.079	3.05±0.91	3.35±1.25	3.26±1.12	0.142

BMI, body mass index; HbA1c, glycated hemoglobin; ALT, alanine aminotransferase; AST, aspartate transaminase. TC, total cholesterol; TG, triglycerides; HDL, high-density lipoprotein; LDL, low-density lipoprotein. *P* < 0.05 were considered statistically significant. Data with P<0.05 are marked in bold for clarity.

**Figure 3:**
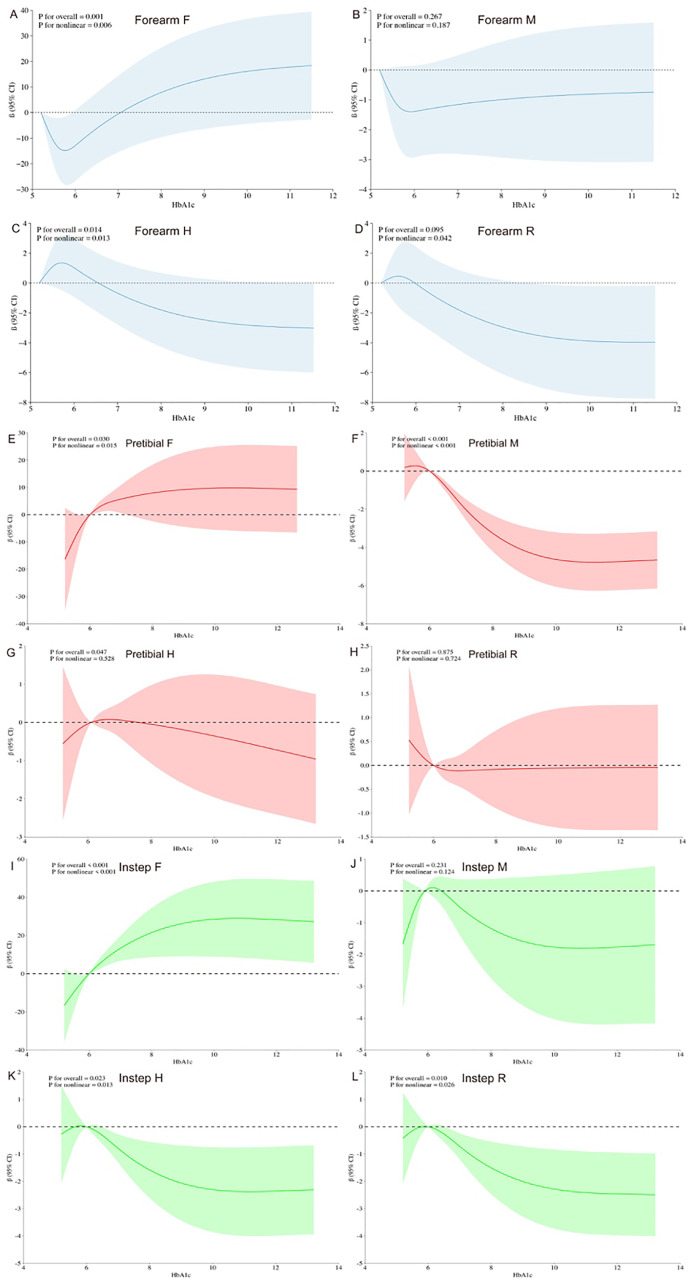
Dose–response relationship of HbA1c levels and biomechanical properties of the skin using a restricted cubic spline. 95% CI, 95% confidence intervals. All models were adjusted for age, sex, BMI, fasting blood glucose, total cholesterol, high-density lipoprotein cholesterol, low-density lipoprotein cholesterol, and triglycerides concentration. Y-axis represents the beta to present biomechanical properties of the human skin for any value of HbA1c.

**Figures 3A–D** illustrates the dose–response relationships of HbA1c with biomechanical properties of the skin at forearm based on RCSs. Specifically, a distinct “J-shaped” association is observed between HbA1c and the forearm F value in the general population (**Figure 3A**, P for overall=0.001, P for nonlinearity= 0.006). Additionally, **Figure 3C** reveals an “Upside-down J-shaped” pattern in the relationship between HbA1c and the forearm H value (**Figure 3C**, P for overall = 0.014, *P* for nonlinearity= 0.013). Furthermore, **Figure 3D** demonstrates a statistically significant nonlinear relationship between HbA1c and the forearm R value (**Figure 3D**, P for nonlinearity=0.042).

**Figures 3E**–**L** revealed a significant nonlinear association between HbA1c and pretibial, instep F value in the general population (**Figure 3E**, P for overall=0.030, *P* for nonlinearity= 0.015; **Figure 3I**, P for overall <0.001, *P* for nonlinear < 0.001). Moreover, **Figure 3F** revealed an “Inverted S-shaped” association between HbA1c and the pretibial M value (**Figure 3F**, P for overall <0.001, *P* for nonlinear < 0.001). In addition, **Figure 3G** underscored a significant negative linear association between HbA1c and the pretibial H value (**Figure 3G**, P for overall = 0.047, P for nonlinear= 0.528). Lastly, **Figures 3K, L** exhibited an “Upside-down J-shaped” relationship between HbA1c and both the instep H and R values. **Figure 3K** demonstrated this pattern for the instep H value (**Figure 3K**, P for overall =0.023, *P* for nonlinear=0.013), while **Figure 3L** showed it for the instep R value (**Figure 3L**, P for overall =0.010, *P* for nonlinear=0.026).

## 4 Discussion

Diabetic skin disorders are one of the most complications of diabetes, yet they are also the most easily overlooked, with an incidence rate of about 51.1% to 97.0% (19). In this study, we quantitatively assessed the biomechanical properties of the skin in patients with T2DM using a non-invasive electro-mechanical device. Our findings revealed that individuals with T2DM exhibited significantly higher F values (indicating increased skin stiffness), and lower M, R, and H values (reflecting reduced elasticity, responsiveness, and resilience) across all measured skin regions compared to healthy controls. These results suggest systemic alterations in skin biomechanical integrity associated with poor glycemic control.

Biomechanical properties are an important theme in skin research. An accurate quantitative assessment of skin biomechanics is useful for characterization of some skin diseases. In the past 15 years, several available devices for measuring the biomechanical behavior and characteristics of human skin have been published, such as SkinFibrometer ^®^, Cutometer ^®^, DUB ^®^ Skinscanner, etc. (20, 21) Most current systems assess skin properties by applying a variable load to the skin surface. Advances in electronics and knowledge have led to the development of new approaches, such as the Khelometer^®^. This device records the force opposing progressive lateral compression, with adjustable amplitude and speed according to the anatomical site being studied. Preliminary studies suggest that the Khelometer^®^ is adaptable to almost all anatomical skin sites. It can measure skin tightness, laxity, hardness, and the degree of collagen aging by analyzing differences in stress response between the skin and the device.

Previous studies have suggested that the level of neutrophil to lymphocyte ratio (NLR) is of great significance in predicting the prognosis of complications of diabetes (22). In addition to predicting through blood indicators, we measure through non-invasive skin elasticity instruments. This study used Khelometer^®^ to elucidate the biomechanical properties of the skin of patients with diabetes. Our research found that alterations in skin biomechanics of diabetes not only occur in the pretibial and feet, but also in the forearm. Diabetes skin changes can be manifested as ulcers in the foot, diabetes like acanthosis nigricans in the neck, and forearm lesions are relatively rare. Previous studies have shown that subcutaneous granuloma annularis (SGA) of the forearm is associated with insulin dependent diabetes (IDDM) (23). Our observations reveal that diabetic patients exhibit significantly higher F values in their forearms, pretibial regions, and insteps when compared to healthy individuals. This finding suggests that diabetes may contribute to an increase in skin hardness. Furthermore, our research indicates that diabetic patients experience more severe skin degeneration in their forearms and insteps, lower skin elasticity in the pretibial region, and poor resilience in the instep skin, as compared to healthy subjects. Our results are similar with Kontter et al. (24) and Periyasamy et al. (25), who observed an increase in skin hardness among individuals with diabetes mellitus and neuropathy. However, we used a different measurement instrument and assessed a wider range of skin sites in our study, as well as having a larger sample size. Furthermore, we divided these individuals into tertiles and found that the group with the high F-value(T3) had significantly higher HbA1c levels compared to the other two groups (T1 and T2). Intriguingly, a similar pattern emerged among individuals with low M, R, and H values (T1), who also showed significantly higher HbA1c levels compared to the remaining two groups (T2 and T3). In our analysis using linear regression to investigate the relationship between HbA1c level and biomechanical properties of the skin, we observe a positive correlation between HbA1c level and skin hardness on the forearms, pretibial, and instep. Moreover, higher levels of HbA1c are associated with increased skin degradation on the forearms and instep, reduced elasticity of the pretibial skin, and diminished resilience of the pretibial and instep skin. Our results revealed that chronic hyperglycemia contributes to the changes of biomechanical properties of the skin. The skin, being the largest organ of the body, is also susceptible to these complications (26). The fragmentation of collagen fibers is a prominent feature of human skin degeneration, which seriously damages the structural integrity and mechanical properties of the skin. Elevated levels of matrix metalloproteinase(MMP)-1 and MMP-2, increased expression of lysyl oxidase (LOX), and heightened crosslinking of collagen in the dermis of diabetic skin contribute to the accumulation of fragmented and crosslinked collagen (13). This process impairs the structural integrity and mechanical properties of dermal collagen in diabetes. Collagen cross-linking makes it difficult for the skin to repair easily, resulting in a decrease in skin resilience and elasticity (27).

Research has shown that due to lower skin temperature, slow blood flow, increased plasma viscosity, and vessel fragility, the skin of pretibial was likely to suffer from diabetes dermatosis (28). Alexandra et al. found that the skin blood flow on the pretibial area of patients with diabetes was slower than that of normal people (29). Sanjay Kalra et al. proposed that diabetes dermatosis is usually related to chronic microvascular and macrovascular dysfunction (30). Kristof Gal et al. found that when the viscosity of whole blood and plasma is reduced through rheopheresis treatment, the ulcer of diabetes foot patients will heal and the pain will be reduced (31). Our research found that alterations in skin biomechanics of diabetes not only occur in the pretibial and feet, but also in the forearm, suggesting that the biomechanical changes in diabetic skin are systemic.

Previous studies have elucidated numerous mechanisms that can result in skin lesions in patients with diabetes. Nevertheless, there remains a notable scarcity of research delving into the relationship between HbA1c levels and the biomechanical properties of diabetic skin. Consequently, we used RCSs to comprehensively explore the influence of HbA1c on the biomechanical properties of skin in diabetic individuals. Our results revealed strong nonlinear associations between HbA1c levels and the biomechanical properties of the skin. Specifically, we observed that as HbA1c levels increased, there were significant alterations in the skin’s elasticity, stiffness, and potentially other mechanical characteristics, indicating a complex interplay between glycemic control and skin integrity in diabetic patients.

T2DM is characterized by a persistent elevation of blood glucose levels, which triggers the onset and advancement of non-enzymatic glycation reactions involving proteins, lipids, and nucleic acids. These glycation processes give rise to a diverse array of chemical entities termed AGEs, which are pivotal in driving the pathophysiological mechanisms underlying diabetic complications (32). AGEs are a group of heterogeneous molecules formed endogenously by non-enzymatic reactions between reducing sugars (including fructose and glucose) and proteins, lipids, or nucleic acids, as well as subsequent chemical rearrangements (33). Because of the high concentration of circulating sugar in the blood, endogenous AGEs formed faster in people with diabetes. The increase in AGEs levels is not solely attributable to type 2 diabetes but may also be associated with obesity. A recent study (34) indicates that elevated AGEs levels are linked to obesity, which is often accompanied by high blood sugar, dyslipidemia, and insulin resistance, all of which contribute to the development of metabolic syndrome and diabetes. In our study, patients had higher BMI levels compared to the control group, which may be attributed to the combined effect of elevated blood sugar and increased AGEs levels. AGEs might lead to the loss of structural collagen, leading to the loss of skin mechanical properties (35). HbA1c is the first type of AGE discovered and is commonly used as a suitable biomarker for detecting average blood sugar levels (5). AGEs induce pathological changes in the skin through three processes. Firstly, AGEs interact with their specific cell receptors, altering the levels of soluble signaling molecules such as cytokines, hormones, and free radicals. Secondly, during non-enzymatic glycation reactions, a large amount of reactive oxygen radicals is released, leading to oxidative stress and a significant reduction in levels of glutathione, Vitamin C, and Vitamin E in the body. This disruption results in synthetic disorders of collagen in skin tissues. Thirdly, AGEs alter the physical and biological properties of the original extracellular matrix proteins, such as collagen (5, 27). Therefore, HbA1c, as an endogenous early glycation product, accumulates and leads to skin aging, characterized by increased hardness, decreased elasticity, reduced resilience, and overall skin degeneration.

Hemodialysis is one of the methods to improve the endothelial dysfunction of Diabetic Foot Disease (DFD) in diabetes foot syndrome. Diabetic foot ulcers are caused by oxidative stress and endothelial inflammation due to high blood sugar. High blood sugar increases the endothelial expression of inflammatory cytokines, Endothelin-1, and endothelin converting enzyme. Through rheopheresis, it can reduce the concentrations of Endothelin-1 and Thromboxane B2 in plasma, improving diabetic foot syndrome (34). Based on our research, inhibition of AGEs may improve diabetes complications such as diabetes skin lesions. AGEs inhibitors include carbonyl trapping agents that reduce carbonyl stress, metal-ion chelators and free radical scavengers that inhibit oxidation, crosslinking breakers that reverse AGEs crosslinking, and compounds that activate the anti-glycation system and Receptor for AGE antagonists (36).

In summary, our study utilized the Khelometer^®^ to quantitatively assess the biomechanical properties of the skin in individuals with T2DM. Notably, we uncovered nonlinear associations between HbA1c levels and these skin biomechanical properties. These novel findings reinforce the existing evidence that elevated HbA1c levels can significantly influence the biomechanical characteristics of the skin in patients with T2DM. The intervention of trained health coaches can reduce HbA1c and further reduce complications of diabetes (37).

Our results align with previous studies (e.g., Kontter et al., Periyasamy et al.), which also demonstrated increased skin stiffness in diabetic patients. However, our study expands on prior work by using a novel device, evaluating multiple anatomical regions, and analyzing dose–response relationships between HbA1c and skin biomechanics. Importantly, we observed non-linear associations between HbA1c and biomechanical indices, suggesting threshold effects or compensatory mechanisms in early vs. advanced stages of glycemic dysregulation.

From a clinical perspective, these findings highlight the potential of skin biomechanical assessment as a noninvasive marker for early detection of diabetic skin complications. Traditional methods such as biopsy or skin autofluorescence require specialized equipment or are invasive. In contrast, the Khelometer^®^ provides real-time, operator-friendly assessments, making it suitable for routine clinical use or monitoring therapeutic responses.

Compared with some previous tools for measuring skin indicators, our method has certain advantages. Abd A Tahrani et al. evaluated DPN using the Michigan Neurological Screening Instrument (MNSI) by performing skin puncture biopsies on the upper and lower leg skin, which undoubtedly caused invasive harm to the subjects (14). Wang X et al. measured skin autofluorescence (SAF) using a DM Scan optical signal detector to detect the accumulation of AGEs. Their approach is more complex and requires specific instruments (15). Unlike earlier methods, our approach is straightforward and entirely non-invasive, ensuring participant safety throughout the process.

Our study has some limitations. Firstly, the sample size was not large enough, which might limit the universality and generalization ability of the results. Secondly, there was a lack of long-term tracking and dynamic observation, and it was impossible to fully understand the changing trend of skin hardness and elasticity in diabetes patients and their relationship with disease progress. Finally, the subjects in our study were all Han Chinese adults. Thus, the results cannot be generalized to other ethnicities.

## 5 Conclusions

The data indicate the electro-mechanical skin device is useful to quantitate the biomechanical properties of the skin with T2DM. Furthermore, this study demonstrates a nonlinear relationship between HbA1c levels and the biomechanical properties of the skin. These findings add to the evidence to support the impact of elevated HbA1c on biomechanical properties in individuals with T2DM.

## Data Availability

The original contributions presented in the study are included in the article/supplementary material. Further inquiries can be directed to the corresponding authors.
